# Case report: Two cases of prostate adenocarcinoma progressing to rare sarcomatoid carcinoma with normal PSA levels following endocrine therapy

**DOI:** 10.3389/fonc.2024.1456390

**Published:** 2024-09-05

**Authors:** Zhicheng Dai, Weikang Wang, Haifang Guan, Xiaohui Wang, Yongheng Ren, Ying Qiu, Jie Liu

**Affiliations:** ^1^ Department of Clinical Medicine, Shandong Second Medical University, Weifang, China; ^2^ Department of Surgical Teaching and Research, Shandong Medical College, Linyi, China; ^3^ Department of Nursing, Shandong Second Medical University, Weifang, China; ^4^ Department of Pathology, Linyi People’s Hospital, Linyi, China; ^5^ Department of Urology, Linyi People’s Hospital, Linyi, China

**Keywords:** prostate, sarcomatoid carcinoma, endocrine therapy, carcinoma, malignant tumor

## Abstract

**Background:**

Patients with prostate adenocarcinoma undergoing regular endocrine therapy may maintain normal PSA levels during follow-up, yet still progress to the highly malignant and rare prostatic sarcomatoid carcinoma, which is seldom reported. This article presents two case studies of prostatic sarcomatoid carcinoma. To date, only a few publications have described prostatic sarcomatoid carcinoma, and the clinical, morphological, and molecular dimensions of prostate adenocarcinoma warrant further investigation.

**Case description:**

Patient A was admitted two years ago due to difficulty urinating, with a PSA level of 6.35 ng/ml. A prostate needle biopsy was performed, and the postoperative pathology diagnosed prostate adenocarcinoma with a Gleason score of 9 (5 + 4, grade group 5). Citing personal reasons, the patient declined a radical prostatectomy and instead received ongoing androgen deprivation therapy (ADT), comprising goserelin, abiraterone, and prednisone. During follow-up, regular PSA tests showed no abnormalities. One year ago, the patient was admitted again due to difficulty urinating and hematuria, choosing to address only the urethral obstruction. Transurethral resection of the prostate was performed, and the postoperative pathology diagnosed sarcomatoid carcinoma of the prostate. Patient B was admitted three years ago due to difficulty urinating accompanied by hematuria. A prostate MRI and a whole-body radionuclide bone scan suggested prostate cancer with bone metastasis. Prostate needle biopsy confirmed the diagnosis. The patient was then regularly treated with androgen deprivation therapy, using goserelin. Throughout the follow-up period, the PSA levels consistently remained within normal limits. One year ago, the patient was admitted due to rectal bleeding. It was speculated that the symptoms of rectal bleeding might have been caused by the prostate cancer invading the rectal wall. A prostate needle biopsy was performed, and the pathology diagnosed sarcomatoid carcinoma of the prostate.

**Conclusions:**

This case underscores the inadequacy of relying solely on PSA levels to monitor high-grade prostate adenocarcinoma during endocrine therapy, as patients may progress to highly malignant atypical variants despite normal PSA levels. We propose that for high-grade prostate cancer patients who are unable to undergo radical prostatectomy, regular and frequent MRI screenings or repeat biopsies should be integral during endocrine therapy and follow-up. Furthermore, a detailed review of the patient’s treatment history and clinical data, including immunohistochemical findings, might offer deeper clinical insights into prostatic sarcomatoid carcinoma.

## 1 Introduction

Prostate sarcomatoid carcinoma, which accounts for less than 1% of all prostate malignancies, is an extremely rare and highly aggressive tumor (1). Research on this disease is currently limited, primarily consisting of case reports and retrospective analyses. A study employed Fluorescence *In Situ* Hybridization (FISH) to confirm that both prostatic sarcomatoid carcinoma and prostatic adenocarcinoma share a common clonal genomic aberration: deletion of the ERG gene (2). This finding indicates that the mesenchymal components in prostate sarcomatoid carcinoma originate from epithelial cells. Consequently, in the 2022 fifth edition of the WHO Classification of Tumors, prostate sarcomatoid carcinoma is categorized as a subtype of acinar adenocarcinoma. It is characterized by the coexistence of malignant epithelial and sarcomatoid components, with the sarcomatoid tissue potentially including spindle cells, smooth muscle sarcoma, angiosarcoma, or cartilaginous and osseous differentiation (3).

## 2 Case representation

### 2.1 Case 1

Patient A, a 71-year-old man visited our hospital for the first time due to symptoms of lower urinary tract obstruction such as frequent urination and difficulty urinating in September 2022, which had persisted for two years. The patient had no history of smoking or alcohol abuse, hereditary diseases, or family history of urinary system tumors. Rectal examination revealed a moderately enlarged prostate gland with a normal texture. No palpable lymphadenopathy was noted, and the rest of his physical examination was unremarkable. His total serum prostate-specific antigen (PSA) level was 6.35 ng/ml, free PSA was 1.00 ng/ml, and neuron-specific enolase (NSE) was 7.55 μg/L. The patient declined further prostate MRI examinations. The preliminary diagnosis was suspected benign prostatic hyperplasia.

**Figure 1:**
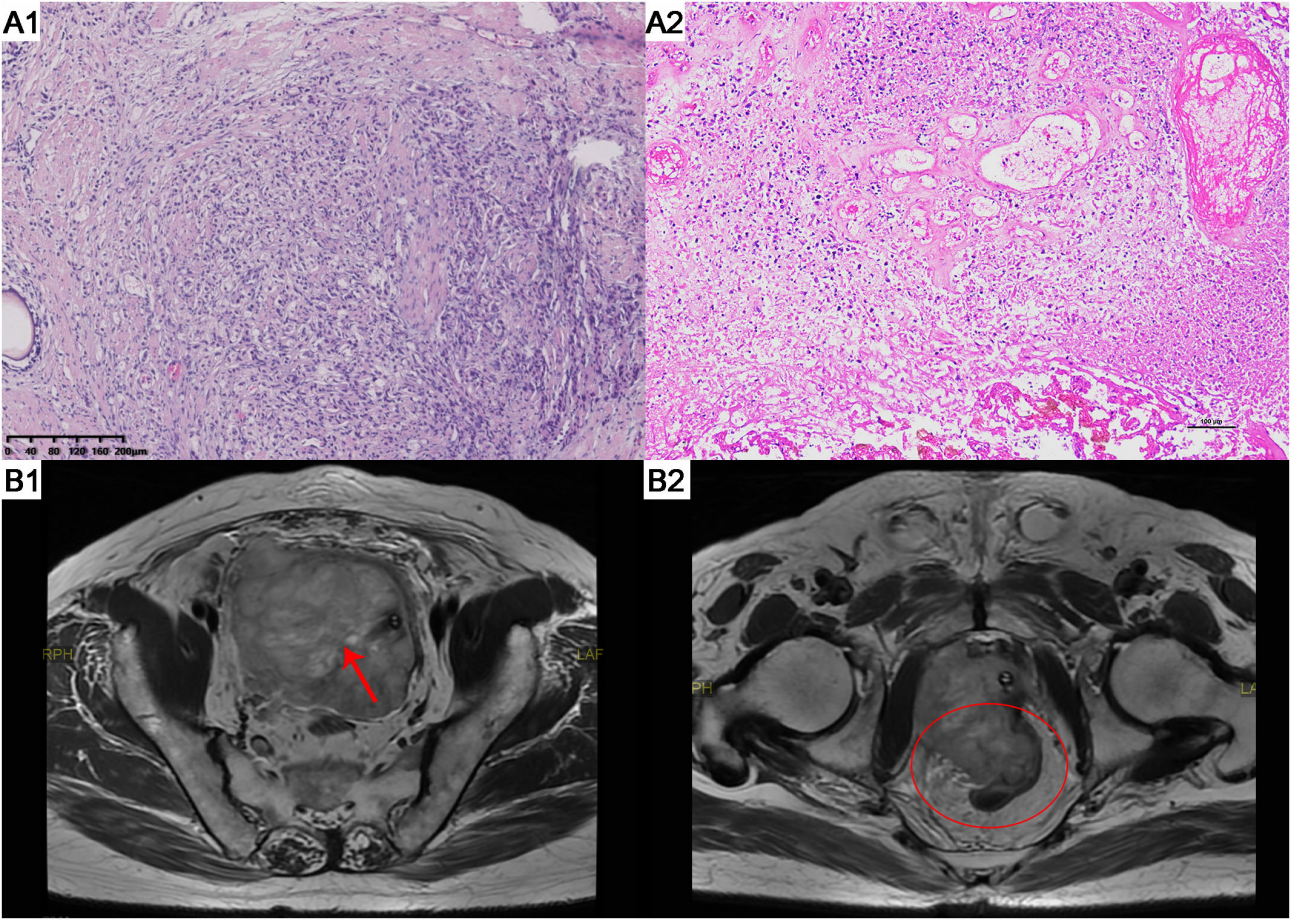
HE staining results and MRI scans of patient A. (A1) The tumor exhibits infiltrative growth. Focally, there are poorly differentiated glandular structures characterized by significant nuclear atypia. The cells are arranged in solid nests or cords. (HE staining, x100). (A2) The tumor cells show significant nuclear atypia, with some cells appearing spindle-shaped. There is a diffuse arrangement of cells accompanied by necrosis. (HE staining, x100). (B1) Prostate MRI showed reveals an enlarged prostate gland with an irregularly shaped, heterogeneous soft tissue mass in the prostate region (Red arrow). (B2) Prostate MRI shows tumor invasion of the rectum (Red oval). HE, hematoxylin and eosin. MRI, magnetic resonance imaging.

**Figure 2:**
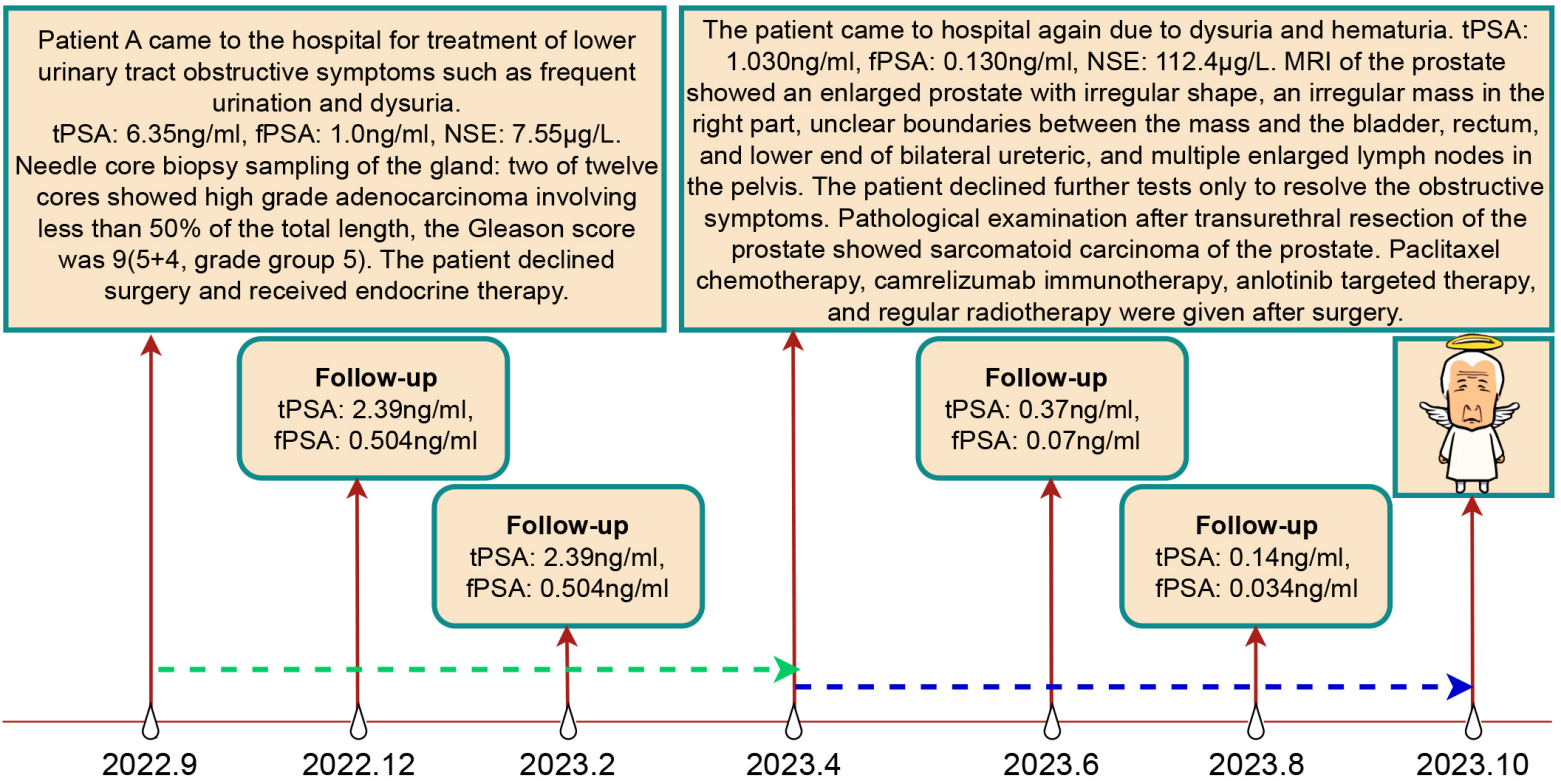
Treatment Course and Follow-up Results of Patient A. The green dashed line represents the endocrine therapy phase, while the blue dashed line represents the non-endocrine therapy phase.

A needle core biopsy of the gland was performed, obtaining twelve cores, two of which showed high-grade adenocarcinoma involving less than 50% of the total length, with a Grade Group 5: Gleason score of 9 (5 + 4) (**Figure 1**). Under the microscope, the cells exhibit significant size and shape variability, indicative of marked cellular heterogeneity. The tumor cell nuclei are deeply stained, large, and irregular in shape, with prominent nucleoli. Nuclear pleomorphism is evident, and the nuclear-cytoplasmic ratio is increased. The cells are arranged in a disordered manner, forming solid sheets or nests, with the glandular structures either obliterated or indistinct. The tissue architecture is disrupted, characterized by tumor cell infiltration and destruction of normal prostatic tissue, with abnormal glandular structures visible. The tumor is monophasic, primarily composed of glandular epithelial cells, with a lack of stromal components. The situation was explained to the patient, and radical prostatectomy was scheduled, but the patient declined for personal reasons. After discussions with the patient, an endocrine treatment regimen (goserelin, abiraterone, and prednisone) was agreed upon, and the patient consented to the treatment plan and further regular follow-ups. The patient’s PSA levels were monitored every three months to assess disease progression. The results during the follow-up period, as shown in **Figure 2**, indicated that PSA levels remained within the normal range.

In April 2023, the patient returned to our hospital with symptoms of difficulty urinating and hematuria. Digital rectal examination revealed an enlarged prostate with a firm texture and a distinct hard nodule. Physical examination showed no obvious abnormalities. Upon admission, laboratory results were as follows: neuron-specific enolase at 112.4 μg/L, total PSA at 1.030 ng/ml, free PSA at 0.130 ng/ml, and testosterone at 9.18 ng/dl. Prostate MRI (**Figure 1**) showed an enlarged prostate with an irregular shape, with an irregular mass on the right side measuring approximately 113mm×82mm. The mass exhibited low signal on T1WI, high signal on T2WI, high signal on DWI, and unevenly low signal on ADC. The mass borders were unclear with the bladder, rectum, and bilateral distal ureters, with multiple enlarged lymph nodes in the pelvic area and band-like high signal on both iliac muscles. Ureteral enhanced CT revealed an irregular soft tissue mass in the prostate area measuring approximately 122mm×79mm with uneven density and marked uneven enhancement. The boundaries with the bladder, rectum, and bilateral distal ureters were unclear, and there were multiple enlarged lymph nodes in the pelvis. The patient refused a whole-body bone scan, expressing a desire only to resolve the urethral obstruction.

**Figure 3:**
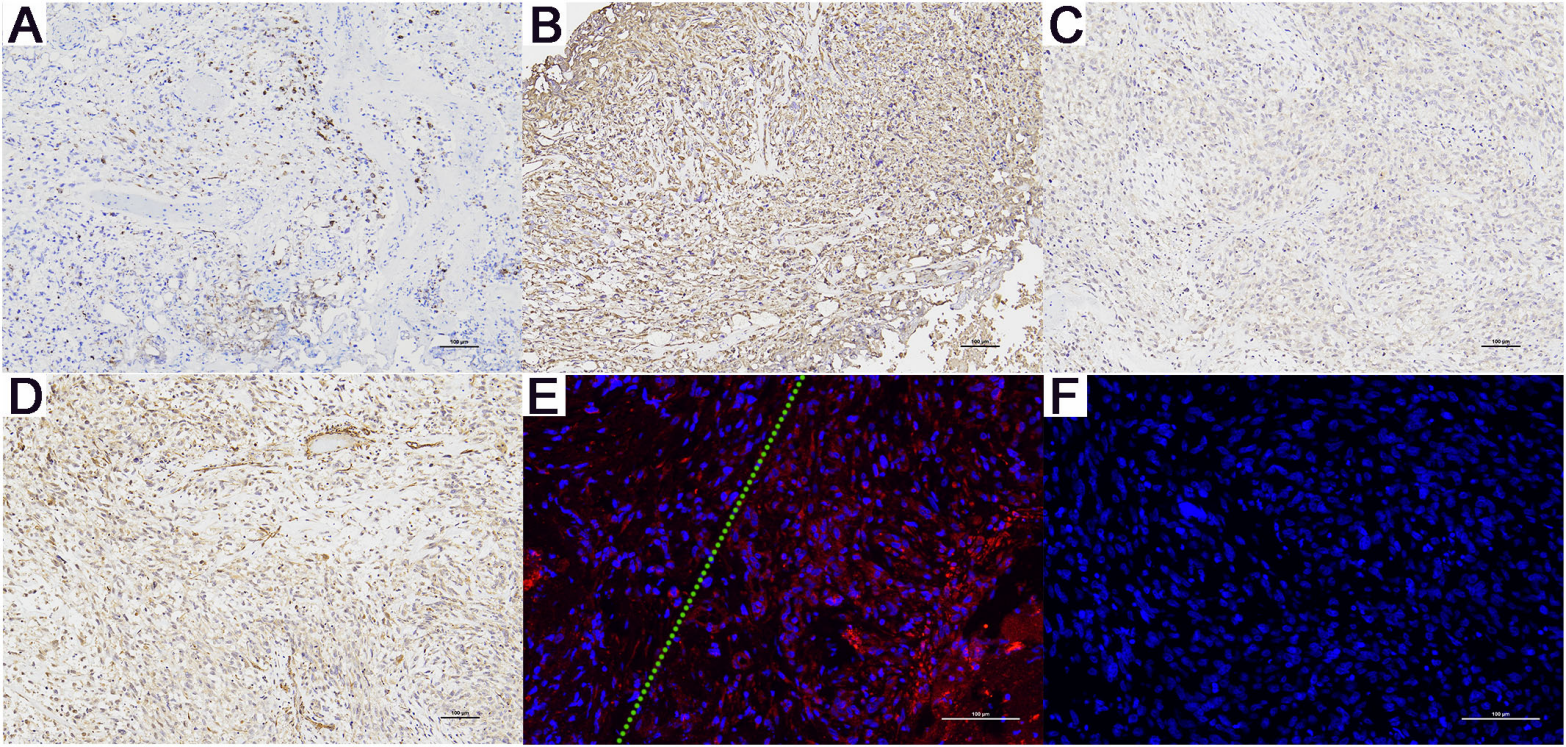
Immunohistochemistry (magnification, x100) and E-Cadherin Immunofluorescence Results (magnification, x200) for Patient A. **(A)** CK (+), only a few cancer cells are positive. **(B)** Vimentin (+), **(C)** AR (+), and **(D)** NES (+). **(E)** Compared to the cells on the right side of the green dashed line, the cancer cells on the left show a significant reduction in E-cadherin expression. **(F)** The negative control for E-Cadherin immunofluorescence. (+): Positive. (-): Negative.

An initial diagnosis of malignant prostate tumor was made. After explaining all conditions and risks to the patient and following his wishes, transurethral resection of the prostate using plasma was performed. During the surgery, a large necrotic tumor was observed in the prostatic part of the urethra, which was friable and bled easily. The bladder neck was extremely narrow with some blood clots seen inside the bladder. Most of the tumor was removed, and the blood clots were washed out of the bladder. Pathological examination (**Figure 1**) revealed that the cells exhibit marked pleomorphism, including spindle and polygonal cells. There is pronounced atypia, with inconsistent cell size, shape, and staining. The nuclei are large and deeply stained, with prominent nucleoli. The cells are arranged in a sarcomatoid pattern, appearing chaotic and disorganized, with indistinct cell boundaries. The tissue architecture is highly disrupted, lacking glandular structures, and extensive necrosis is present. The sarcomatoid component predominates, with a high cell density. The tumor is highly heterogeneous, comprising both epithelial and sarcomatoid elements, demonstrating the characteristics of a biphasic tumor. Immunohistochemical staining (**Figure 3**) showed the following: P504s (-), PSA (-), Vimentin (+), P40 (-), P63 (-), CK7 (-), CK20 (-), CD34 (-), PR (-), CK (partially +), AR (+), and NES (+). Based on the pathology and immunohistochemical findings, the patient was diagnosed with prostatic sarcomatoid carcinoma. Given the potential association between prostatic sarcomatoid carcinoma and Epithelial-Mesenchymal Transition (EMT), we performed immunofluorescent staining for E-cadherin on the resected tissue from this patient. The results showed a reduction in E-cadherin expression in some cancer cells, as seen in **Figure 3**. Postoperatively, the patient was referred to the oncology department for sequential single-drug chemotherapy with paclitaxel, immunotherapy with carrelizumab, targeted therapy with anlotinib, and regular radiation therapy. After discharge, PSA levels were tested every three months and remained normal. Follow-up until October 2023 showed that the patient passed away due to cachexia. The patient’s treatment history and main test results are shown in **Figure 2**.

### 2.2 Case 2

**Figure 4:**
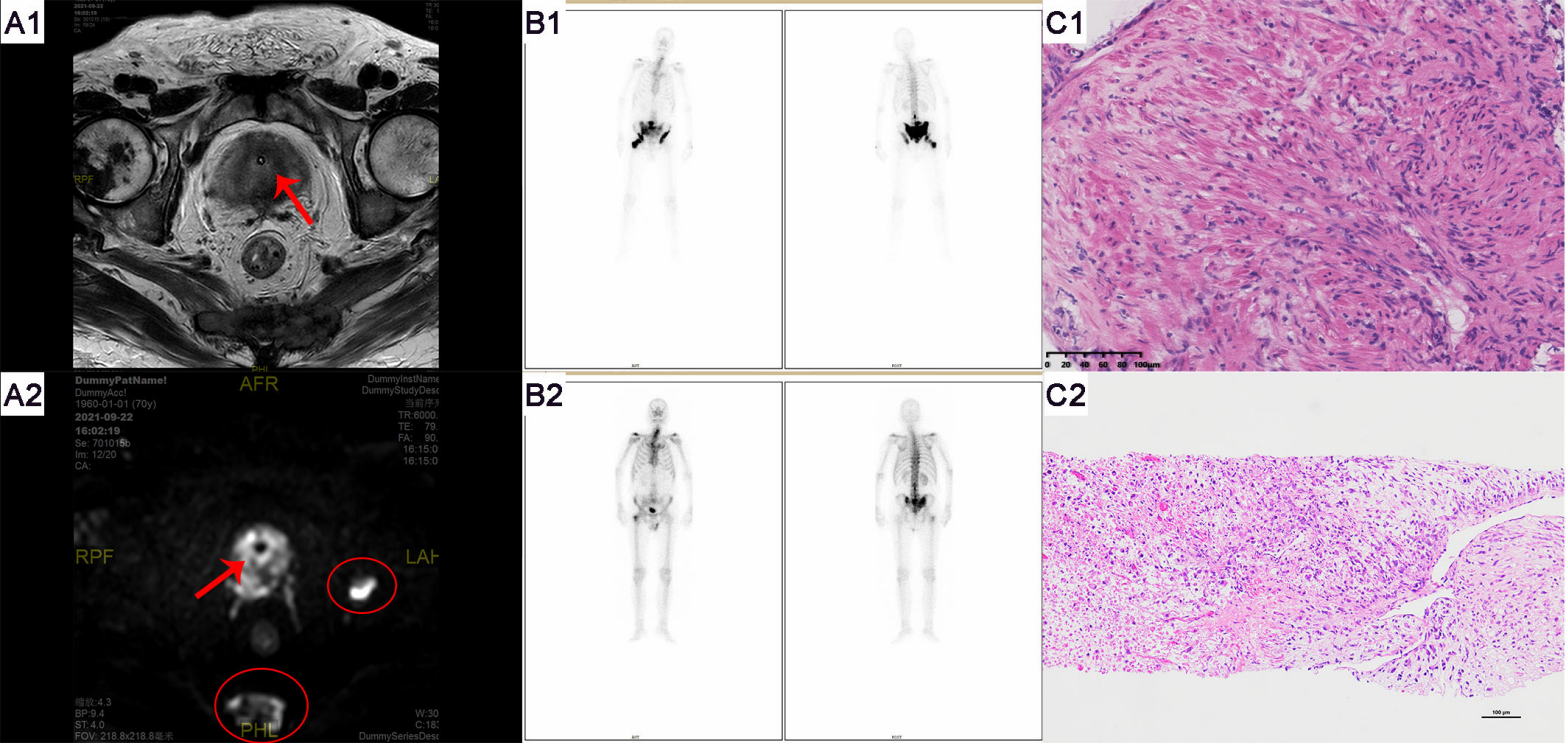
Imaging and pathological examination results for Patient B. **(A1-A2)** MRI of the prostate shows an enlarged gland, especially in the central zone. Bone destruction is observed in both acetabula, the right femoral head, and the sacrum, indicating prostate cancer with multiple bone metastases. Red arrows denote the tumor, and red ovals highlight the bone metastases. **(B1)** Whole-body bone scans from September 2019 (at initial presentation) and **(B2)** May 2023 (post-endocrine therapy). **(C1)** The pathological HE staining from September 2019 shows diffusely arranged spindle-shaped tumor cells with infiltrative growth, significant nuclear atypia, and no apparent glandular structures (magnification, x100). **(C2)** The pathological HE staining from September 2019 shows tumor cells exhibit diverse morphologies, including spindle-shaped and stellate forms, among others. The cells demonstrate a disordered arrangement, lacking clear organizational structure, and display pronounced stromal characteristics. The cells exhibit invasive growth patterns and are accompanied by necrosis (magnification, x100).

Patient B, a 70-year-old male firstly presented to our hospital in August 2021 due to symptoms of difficulty urinating. The patient had no history of smoking or alcohol abuse, no hereditary diseases, and no family history of urinary system tumors. Digital rectal examination revealed an enlarged prostate with a smooth surface, tough texture, clear margins, and a shallow central sulcus. No other significant abnormalities were noted on physical examination. His total serum prostate-specific antigen (PSA) level exceeded 100 ng/ml, and free PSA was above 50 ng/ml. Prostate MRI (**Figure 4**) showed an enlarged prostate with homogenous signal on T1-weighted images and heterogeneously slightly high signal on T2-weighted images, with uneven high signal on diffusion-weighted imaging (DWI); bone destruction was observed in both acetabula and the right femoral head, with low signal on T1 and high signal on fat-suppressed T2-weighted images, suggesting prostate cancer with multiple bone metastases. Whole-body bone scan (**Figure 4**) also indicated multiple bone metastases from prostate cancer. Electrocardiogram, chest CT, and cardiac ultrasound showed no significant abnormalities.

**Figure 5:**
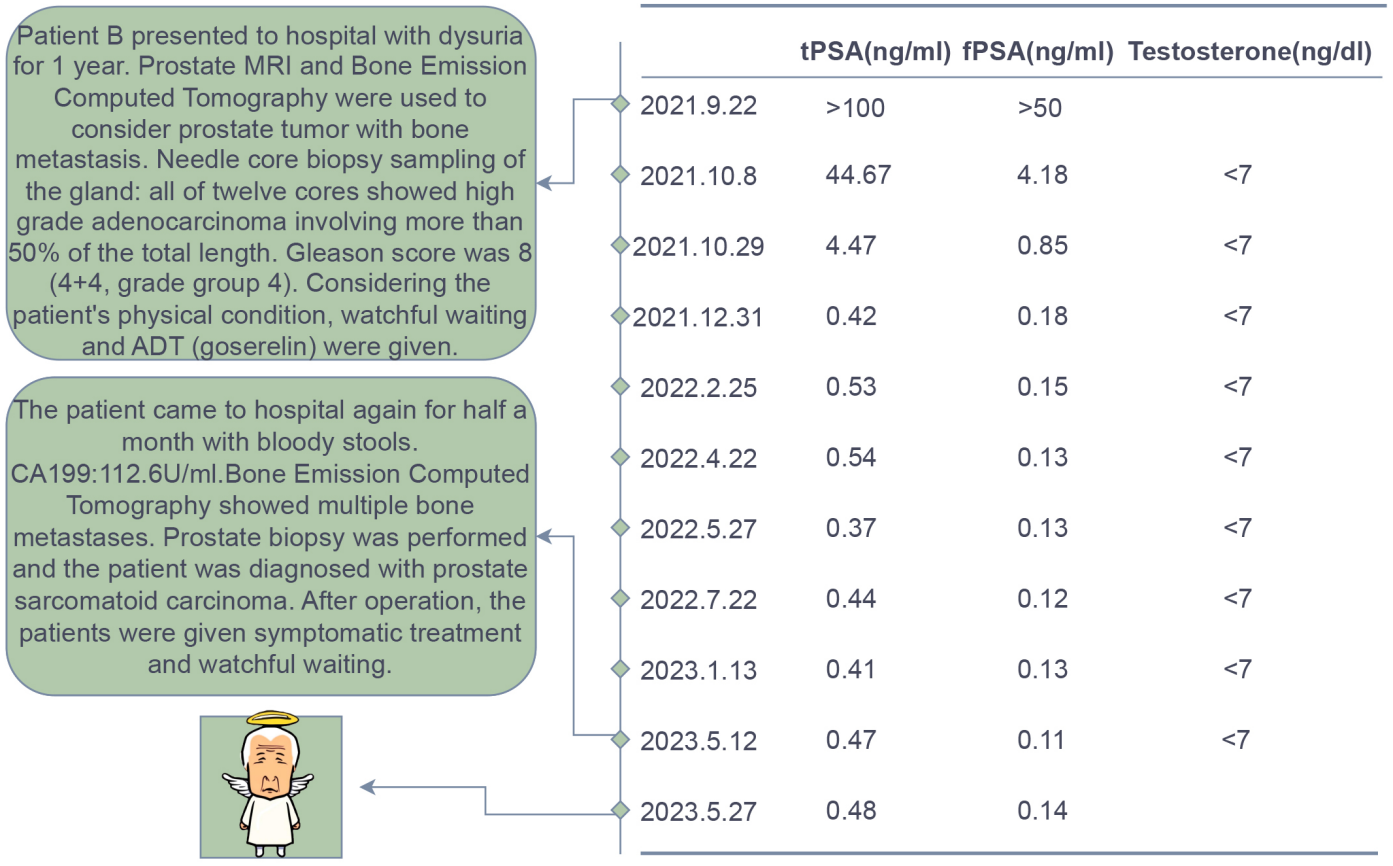
Treatment Course and Follow-up Results of Patient B.

The preliminary diagnosis was a malignant prostate tumor with bone metastases. A prostate needle core biopsy was performed, obtaining twelve cores, all of which showed high-grade adenocarcinoma involving more than 50% of the total length, with a Grade Group 4: Gleason score of 8 (4 + 4) (**Figure 4**). Under the microscope, the tumor cells exhibit considerable variability in size and shape. The cell nuclei are markedly enlarged and deeply stained, with significant nuclear pleomorphism and an increased nuclear-cytoplasmic ratio. Cellular atypia is pronounced, and abnormal mitotic figures are present. The cells are arranged in a disorganized manner, predominantly forming solid sheets or nests. Tumor cells infiltrate normal tissue, with normal prostatic glands obliterated and abnormal glandular structures visible. Some areas show inflammatory cell infiltration. The tumor is monophasic, primarily composed of glandular epithelial cells, with no significant stromal components. Considering the patient’s advanced age and physical condition, the situation was explained to the patient’s family, and watchful waiting and androgen deprivation therapy (ADT) with goserelin was initiated. The patient’s PSA levels were monitored monthly to assess treatment efficacy and disease progression. All PSA results during the follow-up are shown in **Figure 5** and remained within normal ranges. Due to the discrepancy between the patient’s low PSA values and severe early disease condition, further imaging studies were recommended by doctors, but the patient declined.

In May 2023, the patient revisited our hospital due to persistent symptoms of rectal bleeding lasting half a month. Digital rectal examination revealed an enlarged prostate with a smooth surface, firm texture, clear margins, and a shallow central groove. His total serum prostate-specific antigen level was 0.8 ng/ml, free PSA was 0.17 ng/ml, testosterone was below 7 ng/dl, and CA199 was 112.6 U/ml. Whole-body bone scintigraphy (**Figure 4**) showed intense nucleotide distribution in multiple areas including bilateral clavicles, parts of the thoracic vertebrae, parts of bilateral ribs, multiple areas of the pelvis, and the upper segment of the right femur, suggesting widespread bone metastases. Electrocardiogram, chest CT, and cardiac ultrasound showed no significant abnormalities.

**Figure 6:**
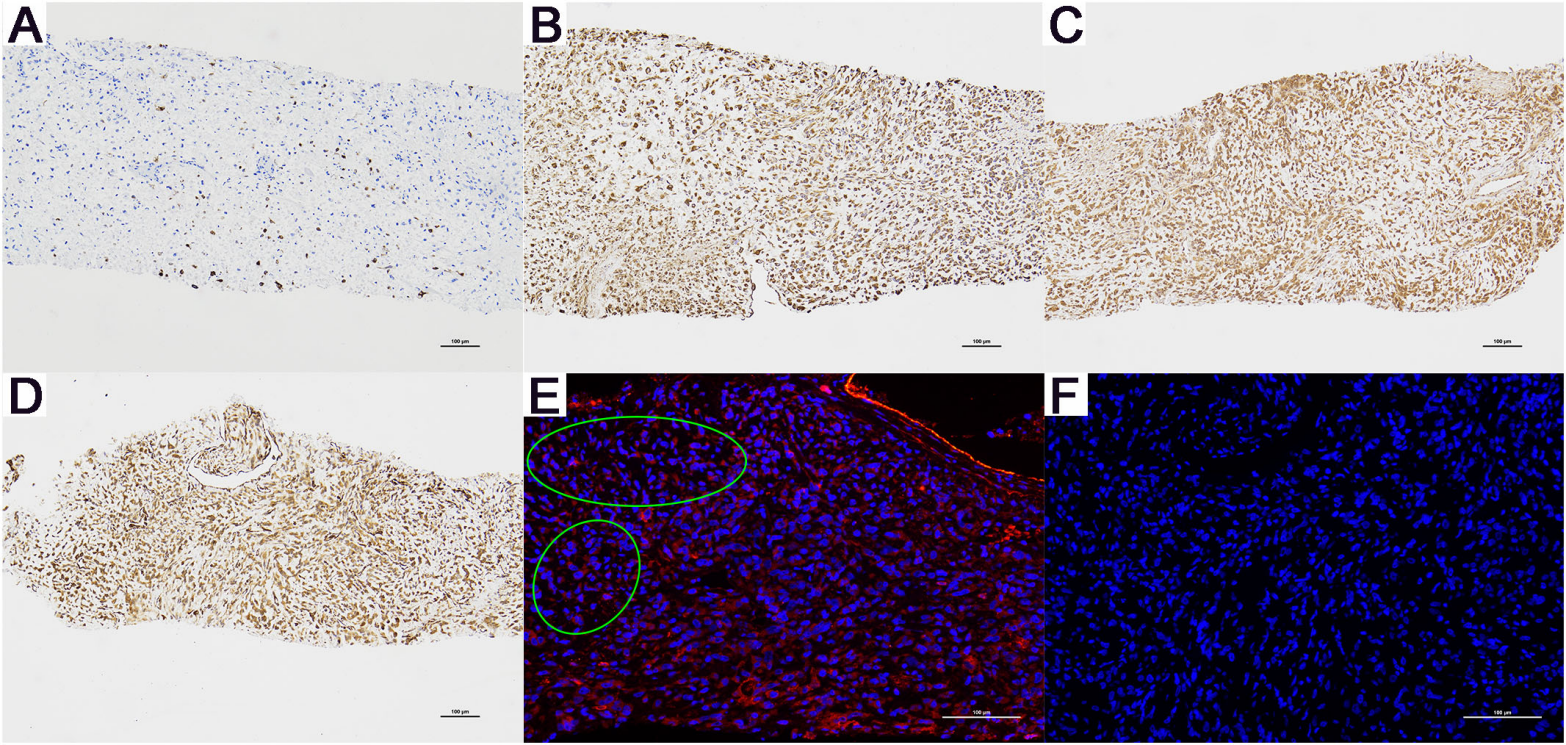
Immunohistochemistry (magnification, x100) and E-Cadherin Immunofluorescence Results (magnification, x200) for Patient B. **(A)** CK (+), only a few cancer cells are positive. **(B)** Vimentin (+), **(C)** AR (-), and **(D)** NES (+). **(E)** Compared to the cells in other areas, the cancer cells within the green ellipse exhibit a significant reduction in E-cadherin expression. **(F)** The negative control for E-Cadherin immunofluorescence. (+): Positive. (-): Negative.

The preliminary diagnosis was malignant prostate tumor with multiple bone metastases, and it was hypothesized that the patient’s symptoms of rectal bleeding could be due to prostate cancer invading the rectal wall. A perineal prostate needle biopsy was performed. Pathological examination (**Figure 4**) showed the cells exhibit considerable variability in size and shape, including spindle and polygonal cells. The nuclei are large and deeply stained, with prominent nucleoli and an increased nuclear-cytoplasmic ratio. There is pronounced atypia, with inconsistent cell size, shape, and staining, and significant pleomorphism. Abnormal mitotic figures are abundant. The cells are arranged in a chaotic manner, with sarcomatoid cells displaying dense, interwoven, or fascicular patterns. The tissue architecture is highly disrupted, dominated by sarcomatoid components, with no normal glandular structures. Sarcomatoid cells are densely packed, and the stroma is abundant. The tumor is biphasic, containing both epithelial and sarcomatoid elements, with high heterogeneity. Extensive areas of necrosis are present. Immunohistochemical staining (**Figure 6**) showed Vimentin (+), AR (-), NES (+), P504s (-), PSA (scattered positive cells), CK (scattered positive cells), and ERG (-). Combining the pathological findings and immunohistochemistry, the disease was diagnosed as prostate sarcomatoid carcinoma. Given the potential association between prostatic sarcomatoid carcinoma and EMT, we performed immunofluorescent staining for E-cadherin on the resected tissue from this patient. The results showed a reduction in E-cadherin expression in some cancer cells, as seen in **Figure 6**. Symptomatic treatment and watchful waiting plan were provided to the patient. As of May 2023, the patient had passed away due to cachexia related to the disease.

## 3 Discussion

PSA, a serine protease, is predominantly secreted by prostate gland epithelial cells (4). Since the 1980s, due to its unique organ specificity, PSA has increasingly become an indispensable marker for detecting prostate cancer. Even today, using PSA to monitor disease status and progression remains a recommendation in the vast majority of guidelines for prostate diseases. However, in cases where patients with prostate adenocarcinoma progress to the rare prostate sarcomatoid carcinoma under regular endocrine therapy without an increase in PSA levels, physicians may be misled into believing the adenocarcinoma is controlled, thereby masking the disease’s actual progression. This case is unique and to the best of our knowledge, no articles have yet described this specific situation.

Prostate sarcomatoid carcinoma is a rare and highly malignant disease characterized by the coexistence of malignant epithelial tissue and malignant stromal tissue derived from epithelial cells (2). The stromal component can include elements such as spindle cells, leiomyosarcoma, angiosarcoma, cartilaginous, or osseous differentiation (3). It is currently understood that the mesenchymal components in sarcomatoid carcinoma originate from epithelial tissues (2), although the specific mechanisms are still unclear. We hypothesize that these mechanisms may involve lineage changes, including epithelial-to-mesenchymal transition (EMT) and/or neuroendocrine differentiation (NED) (5).

Cellular plasticity is a fundamental characteristic of cells. During embryonic development, embryonic stem cells differentiate into mature somatic cells with different physiological functions. However, in pathological states such as injury or disease, mature somatic cells can undergo dedifferentiation and lineage changes (6, 7). Studies have shown that differentiated mature cells can be induced *in vitro* to dedifferentiate into pluripotent stem cells via specific pathways (8, 9). Lineage switching, a fundamental developmental process, enables a single genotype to produce various phenotypes capable of adapting to environmental changes (10). Cancer cells exploit this feature for rampant proliferation and progression.

Epithelial-mesenchymal transition is a critical biological process involved in embryogenesis, tissue repair, and cancer metastasis, characterized by the loss of epithelial traits and acquisition of mesenchymal phenotype. This transformation involves several key mechanisms: loss of cell adhesion, primarily through the downregulation of junctional proteins such as E-cadherin; disruption of apical-basal polarity; reorganization of the cytoskeleton to support motility; and enhanced migratory capabilities (11).The reprogramming of gene expression is centrally coordinated by transcription factors including SNAIL, ZEB, and TWIST families, which suppress epithelial markers and induce mesenchymal characteristics. Signaling pathways such as TGF-β, WNT/β-catenin, and Notch play pivotal roles in modulating these transcription factors and facilitating EMT progression (12).Additionally, interactions with the extracellular matrix through matrix metalloproteases contribute to the physical changes necessary for cell migration. Prostate cancer in an EMT-like state exhibits changes in marker expression, such as E-cadherin and vimentin (13). E-cadherin, a marker typically associated with epithelial cells, tends to decrease during EMT, while vimentin, a mesenchymal marker, increases as cells acquire more mesenchymal features. This shift in marker expression reflects the transformation of tumor cells from an epithelial to a more mesenchymal phenotype, which can enhance their migratory and invasive capabilities.

Neuroendocrine differentiation is a critical adaptive mechanism in various cancers, including prostate, lung, and gastrointestinal tumors, characterized by the acquisition of neuroendocrine features by non-neuroendocrine cells. This process is influenced by a confluence of cellular signaling pathways, notably PI3K/Akt and Ras/MAPK, which activate neuroendocrine-specific transcription factors such as ASCL1 and NeuroD1 (14).These transcription factors play pivotal roles in reprogramming gene expression toward a neuroendocrine phenotype. Additionally, cytokines and growth factors like IL-6 and TGF-β drive NED through autocrine and paracrine signaling, further enhancing the neuroendocrine traits (15). Epigenetic modifications, including changes in DNA methylation and histone configurations, also contribute to the regulation of gene expression essential for NED (14). Neuroendocrine differentiation in prostate cancer typically involves the expression of markers like neuron-specific enolase (NES), chromogranin A (CHGA), P63, and CD56, and usually lacks androgen receptor (AR) expression (16, 17). This pattern indicates a shift from the typical hormone-responsive prostate cancer cells towards a phenotype that is less responsive to hormonal therapy due to the lack of AR. These neuroendocrine cells are generally more aggressive and less differentiated, contributing to poorer clinical outcomes.

The decreased expression of epithelial cell markers (e.g., cytokeratins and E-cadherin) and the upregulated expression of mesenchymal cell markers (vimentin) in both patients provide some pathological evidence for EMT. Additionally, the positive NES results in patient A somewhat support the hypothesis of neuroendocrine differentiation. Research has also found that long-term suppression of the AR pathway may lead to tissue dedifferentiation and changes in the prostate cell lineage towards EMT and/or neuroendocrine differentiation (18). However, this case report offers only preliminary theoretical conjectures based on observational results and does not delve into the underlying mechanisms. Further in-depth cellular experiments are required to explore the pathogenesis of prostatic sarcomatoid carcinoma in greater detail.

Patients with prostatic sarcomatoid carcinoma often present with advanced-stage symptoms, with urinary system obstruction being the most common reason for seeking medical attention (19), including symptoms such as urinary retention and hematuria. Notably, serum levels of prostate-specific antigen (PSA) are often normal in patients with sarcomatoid carcinoma (1), which is significant since PSA levels typically correlate with the progression of malignant prostatic epithelial tumors (4). This anomalous phenomenon in sarcomatoid carcinoma patients seems to contradict the common understanding that PSA is the most sensitive biomarker for prostate cancer, thereby complicating disease monitoring. This discrepancy could be attributed to the dedifferentiation of cancer cells, which results in a reduced ability to produce and secrete PSA. Dan et al. (20) reported on 46 prostate cancer patients who progressed despite low PSA levels. Their findings align with the hypothesis presented in this paper and they summarized the characteristics of such patients: 1) Gleason scores >7, 2) atypical histologic variants, 3) locally advanced tumors.

Following the initial diagnosis of high-grade acinar adenocarcinoma in the first patient, active surveillance was considered as a treatment option, in accordance with AUA guidelines (21). However, given the patient’s incomplete diagnostic workup and the general guideline recommendation against surveillance for patients with a Gleason score greater than 6 (22), endocrine therapy was ultimately chosen. This decision does not preclude reflection on this case. Had we opted solely for active surveillance (monitoring PSA every three months, DRE every six months, and annual re-biopsy or prostate MRI), the patient’s outcome could have been worse. The crucial insight from this case is that for patients with high-grade adenocarcinoma under active surveillance, in addition to regular PSA testing, frequent prostate MRI scans are necessary, and repeated biopsies should be performed if MRI results are suspicious. We recommend an MRI frequency of at least every three months and no more than every six months, due to the risk of high-grade adenocarcinoma progressing into highly malignant and rapidly evolving atypical variants, rendering PSA tests ineffective. Of course, radical prostatectomy remains the best option for patients with localized adenocarcinoma (23). The lesson from the second patient in this case is that if PSA levels are exceptionally low during follow-up for a patient with a previously severe prognosis of high-grade prostate cancer, it is crucial to perform imaging studies or repeat biopsies to be vigilant about the potential progression to highly malignant atypical lesions.

Traditional prostate cancer detection methods such as PSA testing, MRI, and tissue biopsy, though essential, have notable limitations. PSA testing’s high false-positive rate leads to unnecessary follow-ups and treatments, causing psychological and financial stress. MRI, while sensitive and specific in tumor localization, is prohibitively expensive. Tissue biopsy, the gold standard for diagnosis, provides detailed histological information but is invasive, with risks of infection and bleeding, and only offers localized tumor data, failing to capture the disease’s systemic progression. The identification of early molecular markers of metastasis and monitoring treatment efficacy are crucial in modern translational research. Liquid biopsy (LB) has emerged as a promising non-invasive detection method, gaining attention for its ability to analyze circulating tumor cells (CTCs), circulating tumor DNA (ctDNA), RNA, and exosomes from bodily fluids (e.g., blood, urine, saliva). This approach offers a more convenient, comprehensive, and dynamic method for cancer detection and monitoring (24). The tumor components most commonly obtained from liquid biopsy and used as biomarkers include extracellular vesicles (EVs), non-coding RNAs (ncRNAs), and CTCs (25). CTCs are an effective prognostic and predictive biomarker for prostate cancer. Patients with five or more CTCs in 7.5 ml of blood typically have a poor prognosis, while those with fewer than four CTCs generally have a better prognosis (26). Non-coding RNAs, specifically long non-coding RNAs (lncRNAs) longer than 200 nucleotides, have also been identified as significant biomarkers in prostate cancer. Studies have highlighted upregulated lncRNA biomarkers such as SChLAP1, PCA3, SPRY4-IT1, PCATs, and TRPM2-AS (27). MicroRNAs (miRNAs), approximately 22 nucleotides in length, are another subtype of ncRNAs relevant in prostate cancer. A recent study using miRNA sequencing and RT-PCR on urinary exosomes (UE) of prostate cancer patients found significant downregulation of Exos-miR-375 and upregulation of Exos-miR-451a, Exos-miR-486-3p, and Exos-miR-486-5p (28). The downregulation of Exos-miR-375 was correlated with clinical T stage and bone metastases. Additionally, miRNA profiling in urine biopsies distinguished benign prostatic hyperplasia (BPH) from prostate cancer, with miR-222-3p, miR-24-3p, and miR-30c-5p being particularly effective (29). Overall, CTCs and ncRNAs are clinically significant biomarkers for diagnosing prostate cancer and monitoring disease development and aggressiveness, offering a promising future for non-invasive cancer diagnostics.

Preoperatively, prostate sarcomatoid carcinoma can be challenging to differentiate from other types of prostate cancer due to its atypical clinical symptoms and nonspecific radiological findings. Postoperative pathological examination and immunohistochemical staining are crucial for differential diagnosis. This disease should be distinguished from prostate carcinosarcoma, sarcomatoid urothelial carcinoma, and acinar adenocarcinoma of the prostate. Pathology can differentiate it from acinar adenocarcinoma and sarcomatoid urothelial carcinoma. Histologically, carcinosarcoma and sarcomatoid carcinoma present similarly, both featuring epithelial and sarcomatoid mesenchymal components. The hallmark of sarcomatoid carcinoma is the presence of malignant epithelial components and sarcomatoid stroma originating from epithelial tissues. In most cases, the sarcomatoid tissue is predominantly composed of spindle cells, although some cases may include leiomyosarcoma, angiosarcoma, osteosarcoma, and chondrosarcoma (30, 31). Carcinosarcoma is a biphasic tumor that contains both adenocarcinoma originating from epithelial tissue and sarcoma arising from mesenchymal connective tissue within a single tumor. In sarcomatoid carcinoma, the spindle cells express vimentin and epithelial tissue markers such as cytokeratin (CK) (32), whereas in carcinosarcoma, the sarcomatous component does not express CK. Additionally, androgen receptor (AR) and prostate-specific antigen (PSA) are typically negative in sarcomatoid carcinoma. The use of fluorescence *in situ* hybridization (FISH) to detect ERG translocation can provide valuable diagnostic insights (2).

There are few reports on the treatment of prostatic sarcomatoid carcinoma, and the prognosis for patients is generally poor. Current evidence does not conclusively demonstrate that surgical interventions improve survival rates, yet surgery remains the preferred treatment. According to a retrospective review by Mark et al. (1), the median overall survival (OS) for 45 patients was 10.6 months (95% CI: 7.16, 19.38) following a median follow-up of 106 months. Survival data from 27 patients were stratified into four groups based on confirmed clinical staging and treatment data: local disease (n=9, median OS not reached after 106 months), local disease with bladder invasion (n=9, median OS: 9 months), metastatic disease (n=6, median OS: 7.1 months), and unstaged disease (n=3, all of whom died shortly after diagnosis without treatment). This suggests that patients with localized sarcomatoid carcinoma undergoing surgery and/or radiation therapy may achieve an overall survival of over five years, indicating the potential efficacy of these treatments for clinically localized disease. In contrast, the prognosis remains poor for patients with local disease plus bladder invasion and for those with metastatic disease, irrespective of the treatment modality. Additionally, the study noted that none of the four patients treated with androgen deprivation therapy (ADT) showed PSA or radiological responses. The similar experiences of two patients in this case, combined with their negative PSA immunohistochemical staining results, suggest that androgen deprivation therapy (ADT) may be ineffective for this disease. This observation appears to corroborate our preliminary hypothesis regarding the disease mechanism proposed earlier, suggesting that sarcomatoid carcinoma may undergo lineage changes and does not rely on androgen receptor signaling.

Furthermore, a study by Donna et al. (31) involving 42 patients with prostatic sarcomatoid carcinoma found that most developed metastases and died within a year of diagnosis. The study also found no correlation between the prior grade of acinar adenocarcinoma and patient survival. Although reports suggest sarcomatoid carcinoma may arise post-radiotherapy in high-grade acinar adenocarcinomas (31, 33), a clear link has not yet been established, necessitating further extensive case studies and statistical validation.

## 4 Conclusions

In summary, prostatic sarcomatoid carcinoma, arising secondary to acinar adenocarcinoma, is extremely rare and highly malignant. It progresses rapidly and is associated with a very poor prognosis. This disease lacks specific clinical symptoms and radiological signs. Patients typically present with normal PSA levels, which makes postoperative pathology and immunohistochemical staining crucial for differential diagnosis. Currently, surgical treatment is the primary approach; early diagnosis and treatment are crucial for improving patient survival rates. For patients with high-grade acinar adenocarcinomas under active surveillance, the interval between prostate MRI screenings should be shortened to ideally every three to six months. This is due to high-grade adenocarcinomas potentially progressing into highly malignant atypical variants, rendering PSA tests ineffective.

## Data Availability

The original contributions presented in the study are included in the article/supplementary material. Further inquiries can be directed to the corresponding author.
